# Impact of indication changes on scoping for European Union Joint Clinical Assessment: scale of the problem and how to address it

**DOI:** 10.1017/S0266462324004641

**Published:** 2024-11-25

**Authors:** Inka Heikkinen, Melinda Goodall, Natalie Steck, Maria Poulakou, Katherine Piso

**Affiliations:** 1 Global Regulatory Policy and Intelligence, MSD Danmark, Copenhagen, Denmark; 2 Goodall HTA Consulting Ltd., UK; 3 Global Access Strategy and Policy, MSD International, Zurich, Switzerland; 4 Regulatory Affairs, MSD Greece, Athens, Greece

**Keywords:** EU HTA, therapeutic indication statement, PICO scoping, Joint Clinical Assessment

## Abstract

**Objectives:**

The European Union Joint Clinical Assessment (JCA) process aligns with the regulatory process to promote faster patient access. The PICO (population, intervention, comparator, and outcome) scoping for the JCA must occur before the regulatory process concludes. The risk of indication change during this period is one of the concerns for the success of the JCA process. We investigated the frequency and type of changes that are made to proposed indications and examined how such changes could impact the PICO scoping for JCA.

**Methods:**

Twenty-seven recently approved oncology and 15 Advanced Therapy Medicinal Products (ATMP) products were included. Observed indication changes were categorized into editorial or population changes population changes were graded based on the anticipated impact on JCA scope depending on their nature.

**Results:**

The majority of products had only editorial changes between proposed and approved indications (67 percent). Once amended, it was common for the indicated population to be narrowed, and rare for it to be broadened. The most common change observed was the shift to a later treatment line. The greatest risk for PICO rescoping would be when new populations would have been added, or new subpopulations or subgroups would have been omitted from the initial scope.

**Conclusion:**

The impact on JCA scope depends on the proposed indication wording and how the PICO scoping would have been conducted. Rescoping warrants a considered decision, and to mitigate the risk of delays, dialogue between the assessors and the developer is recommended for informed decision-making.

## Introduction

In 2021, the European Union (EU) adopted the Regulation on Health Technology Assessment (EU HTAR), which introduces a framework enabling work sharing between HTA bodies through Joint Clinical Assessment (JCA). The overall aim is to reduce duplication of efforts, strengthen HTA quality, and foster faster patient access and sustainable long-term HTA cooperation across the EU [1].

The JCA process is linked to the procedural timetables of the EU centralized marketing authorization process (Supplementary Figure 1) [2]. Key milestones for the JCA process, according to HTAR and the adopted Commission Implementing Regulation [3], and the respective timepoint in the regulatory process, are:

1. The JCA scoping which starts, when the regulatory review process starts, with a PICO (**p**opulation, **i**ndication, **c**omparator, and **o**utcomes) survey being sent to all EU Member States.
2. The JCA dossier submission by the health technology developer (HTD), which takes place 100 days after receiving the adopted assessment scope, however at the latest by 45 days before the opinion from the Committee for Human Medicinal Products (CHMP) of the European Medicines Agency (EMA) is finalized. While this could in principle be during the second clock stop after day 180 of the regulatory review process, due to uncertainty whether there will be a second clock stop and its length (that is will the regulators request more information and how long it will take for the applicant to respond), companies are forced to submit latest at day 165. Otherwise, there is a risk of not meeting the legal deadline.
3. The JCA report generation and publication, which takes place 30 days after the Commission Decision granting marketing authorization.

The proposed therapeutic indication plays a central role in the JCA assessment scope because it defines the target population for whom the benefit–risk balance is expected to be positive and thus, defines the starting point for the PICO survey. The final wording is subject to the regulatory assessment process and should be justified scientifically in the benefit–risk section of the CHMP assessment report and therefore usually reflects the clinical trial evidence [4]. The inclusion and exclusion criteria for patients in clinical studies, patient characteristics, as well as the prespecified disease-relevant stratification factors and subgroups are considered for the indication wording. Regulatory bodies may interpret the clinical trial findings differently, which may in some cases lead to diverging regulatory decisions or approved indications in different jurisdictions [5]. The deliberative process for the indication is reflective of the proposed indication and supporting evidence and is an ongoing scientific dialogue between the regulators and the developer until the CHMP has adopted its opinion.

The JCA assessment scope determines the content of the dossier and informs all subsequent activities. It is important that the final indication is well reflected in the PICO survey to ensure the JCA report can be used in reimbursement decisions at the national level [6]. The legal framework for the HTAR dictates that the assessment scope as well as the dossier generation and submission will take place before the final therapeutic indication has been adopted by the CHMP, incorporating the inherent uncertainty of the final indication. This has been indicated as one of the most challenging areas to navigate successfully in the future system [7]. The first exercises to test the PICO survey by the HTA bodies resulted in up to 6 populations, 11 comparators and 15 outcomes [8–10]. In a similar exercise, the pharmaceutical industry came to up to 10 populations and up to 23 comparators in their selected case studies [11]. The European Commission has clearly indicated its intention to reduce the number of PICOs to the lowest level agreeable to reducing the workload for both the applicants and the JCA assessors, while still meeting the needs of all Members States.

The JCA scoping activity starts with the proposed indication from the HTD. The final scope is reviewed, with consideration of the first list of questions (LoQ) from the regulators at day 120. The assumption is the number of PICOs could be reduced based on the list of questions as a predictor of the population included in the final indication. During the early phase of regulatory review, the rapporteur and corapporteur assess the proposed indication against the evidence provided and may independently propose changes in their Day 80 rapporteur assessment [12]. If an amendment to the indication is proposed or there are questions on the submitted evidence, the HTD can argue against the amendment in their responses to this LoQ. While the details of deliberation processes on indications amongst European regulators are not available in the public domain [13], it is understood that the regulators pose questions on the evidence supporting the proposed indication for every marketing authorization application.

Potential changes to the therapeutic indication during the regulatory assessment process have been a subject of major concern and discussion among HTA bodies particularly in terms of the JCA scoping process [7]. The adopted Implementing Regulation on the JCA procedure outlines in Article 16 the consequences of therapeutic indication(s) changes. The JCA Subgroup would decide if the JCA process could be continued or should revert back to the starting point. If the process is reverted, it would trigger a new revised PICO scoping process, the development and submission of a revised JCA dossier and, (depending on when this happens), the generation of a revised JCA report [3]. Such a “scenario of reversion” would lead to significant delays in the JCA process at EU level, and at the national level as Member States are obligated to take it under due to consideration in national HTA decision-making [2, 14]. This thereby would delay patient access, the opposite of one of the goals of the new regulation.

We therefore wanted to understand how often indications change, how these changes may impact the JCA scoping process, and explore alternative management processes to mitigate delays in the EU level process. It is critical that the new process enables timely and predictable national appraisal processes and ultimately access to new medicines for patients.

## Methods

### Product Identification for the Study

We included all oncology and advanced therapy medicinal products (ATMPs) with new active substances (NAS) that recently underwent the European Medicines Agency’s (EMA) Marketing Authorization assessment procedure in our study because those product categories will be the first to enter the new JCA process. The sample size was gathered from the time periods between 2017 and January 2024 in the case of ATMPs. For NAS in oncology, the period is 2020 and January 2024. Of note, ATMPs included all therapeutic areas to have the biggest possible sample size, while for oncology the most recent approvals were considered most representative. Generics and Biosimilars have not been included as they are out of scope for EU HTAR. Also, conditionally approved products have not been considered because we aimed to investigate a standard situation as it would apply to the majority of new products. Recently new regulatory approvals were identified from the Committee of Advanced Therapies (CAT) quarterly highlights and the approved ATMPs report [16] and through the EMA Human Medicines highlights monthly newsletters [15] respectively. Through the CAT quarterly highlights, we identified 15 ATMPs that were most recently approved by the EMA between July 2017 and January 2024. After the redaction of generics, biosimilars, conditional approvals, and duplicates for ATMPs, we identified twenty-seven oncology products that were approved as NAS by the EMA between November 2020 and January 2024 from the EMA monthly newsletter. The period was considered suitable to provide representative insights into the patterns related to indication changes. The proposed and final approved indications were sourced from the respective European Public Assessment Report (EPAR) published under the Assessment history section on the EMA Web site for each product.

### Categorization of Types of Indication Changes and Applied Definitions

The proposed and final indication wording were compared, and where indication wording differences were identified, changes were categorized as “wording clarification” or “change to eligible population”. To understand the way in which indications change, the changes observed were grouped into the four following themes: “specific prior therapy’, “focused population”, “later treatment line”, and/or “population expanded”. Individual products could be allocated to more than one theme due to the fact that some indications were found to change in more than one way. To conduct a risk assessment for the PICO, we first captured how the indication change could impact the resulting eligible population in clinical practice, and secondly, assessed the associated risk of the specified change for the PICO and JCA. The themes for how indications changed had captured ***what*** the change had been. Therefore, in order to explore ***how*** the resulting population would be impacted, the products with indication changes underwent a second review. For each product, it was captured whether the indication change was expected to add or remove a subgroup or subpopulation in clinical practice. This was based on broad clinical assumptions without expert clinical validation. To estimate the significance of the indication, change to the JCA decision, the expected relative importance of the subgroup or subpopulation to the JCA assessment was also captured. The following definitions were used:

- **Central population or central subgroup:** A population or subgroup that is either the most significant or has very high significance to the decision-making and product value. This may be due to relative population size, level of unmet need, or clinical effectiveness.
- **Subpopulation:** A population that would be expected to have a dedicated PICO and therefore an independent dossier chapter in the JCA. Expected to be supported by evidence from a full data set or prespecified subgroup.
- **Subgroup:** Defined as a population that sits within another population, and therefore within the PICO of a larger group. It would not have its own dossier chapter. It may be considered exploratory, and supported by evidence that requires subgroup analyses that may not have been prespecified.

### Analyzing the Impact to the PICOs and JCA Scope

To assess the risk for the change to the PICO and JCA, four risk categories were defined based on the potential consequences to the PICO and dossier development: major impact, high impact, moderate impact, and minor impact (see Table 1). A risk category was assigned for each of the potential subgroup/subpopulation changes that could occur.

**Table 1.** Risk assessment and categorization of PICO changes

Risk status	Mapped to population impact	Risk and considerations assumed
Major impact	**+** Adding a central subpopulation or central subgroup	Added population/subgroup would not have been included in the original PICO, or not in sufficient detail. Added population/subgroup may require data sources or analyses not readily available to allow for timely and/or complete inclusion in the dossier.
High impact	**+** Adding a subpopulation or subgroup	Added population/subgroup would not have been included in the original PICO, or not in sufficient detail. The additional data needed could have been anticipated in advance or could be easily extrapolated from the current evidence base. Added population/subgroup is a lower priority for decision, and access to it may not warrant delay to the central population – it may be appropriate to proceed without it, or stagger processes.
Moderate impact	– Excluding a central population or central subgroup	The population/subgroup being removed was predefined in the PICO but it may not be straightforward to remove. Including evidence that is no longer relevant would add ambiguity or inappropriate uncertainty to the dossier. It therefore needs to be removed.
Minor impact	– Excluding a subpopulation or subgroup	The populations being removed were predefined in the PICO and can be removed easily. Including the respective evidence would not add ambiguity or uncertainty to the dossier, so it is not essential to remove it.

For each product the expected subgroup/subpopulation change was used to assign a risk category, applying the highest risk of the changes that were expected to be observed. A conservative approach was taken, assuming a worst-case for subjective scenarios.

For indication changes associated with a risk of a major impact – the rationale applied by the regulator was qualitatively analyzed based on the discussion on benefit–risk and overview of clinical efficacy sections in the EPAR to identify patterns, as proposed by Bujar et al. [13]. The potential PICO impact was qualitatively analyzed to consider in which situations and conditions a new scoping process could be warranted.

## Results

### Frequency of Indication Changes

**Figure 1.**
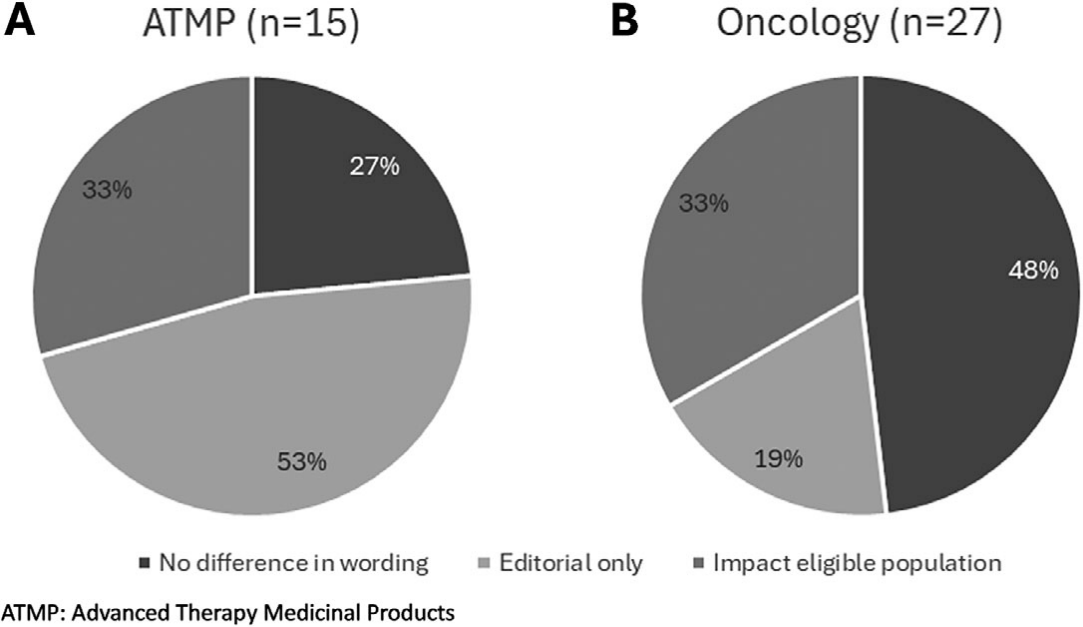
Proportions of the type of changes in the final indication compared to the proposed indication.

For majority of products (67 percent), there were no differences, or only editorial differences, between the proposed and approved indications (Figure 1). Of the 27 oncology products reviewed, 9 had changes that would impact the eligible population. Of the 15 ATMP products reviewed, 5 had changes that would impact the eligible population, of which 2 were ATMP products for nononcologic indications.

For the products that had indication changes (*n* = 14), the majority reduced the eligible population (*n* = 13) and it was rare for the eligible population to be broadened (*n* = 1, an ATMP nononcologic product) (Table 2). The indication changes that reduced the eligible population did so by incorporating treatment-based conditions, such as specifying a prior therapy (*n* = 4) or proposing a later treatment line (*n* = 7), or incorporating patient-based conditions, such as narrowing to a specific patient group (*n* = 7).

**Table 2.** Indication change theme identified for each product with indication changes on the eligible patient population

	Specific prior therapy	Focused population	Later treatment line	Population expanded
**Oncology products**				
Tevimbra	✓			
Orserdu	✓	✓		
Opdualag		✓		
Tepmetko			✓	
Rybrevant		✓		
Trodelvy		✓		
Qinlock	✓		✓	
Nexpovio			✓	
Copiktra		✓	✓	
**ATMP products**				
Spherox (nononcology)		✓		
Yescarta		✓	✓	
Kymriah			✓	
Luxturna (nononcology)				✓
Tecartus	✓		✓	

### Regulator Rationale for Indication Change

Patterns were identified in the regulators’ rationale for each change type (Table 3). The restrictions to indication often matched the provided clinical evidence, particularly evidence from pivotal trials. These would usually relate to the magnitude of the effect on different subgroups of the population (specifically in oncology) and/or the characteristics of the patients involved in clinical trials, particularly in situations where the trials had included patients with a specific prior therapy or a specific genetic profile and/or disorder. For ATMPs, safety was a more prominent reason for proposing a later treatment line, as many cell and gene therapies may have severe side effects, such as cytokine storms for CAR-T therapies. For population expansion, the method of action was generalizable to a larger population with an unmet medical need as the condition is caused by the same disease pathology (gene expression), and the scientific rationale supported expanding the population for all patients with that specific pathology as their benefit–risk profile would be expected to be positive.

**Table 3.** Regulators’ rationale for indication change based on the public assessment report

Type of change	Regulators’ rationale for change
Specifying prior therapy	May be added in case of a consideration that treatment effect follows from exposure to a certain prior treatment. Often follows the inclusion criteria in the pivotal trials (e.g., the studied populations have all received a certain prior therapy).
Restricting the population to a subgroup or a subpopulation	One subgroup demonstrated clear, statistically significant benefit, while either the central population or another subgroup may not have a clear difference in efficacy. Observed in situations of: An internal inconsistency on efficacy and magnitude of the treatment effect among subgroups; or Clinical benefit of the therapy or improvement in an established surrogate end point for clinical benefit has not been made plausible/demonstrated in specific subgroups. Given that the added benefit appeared dependent on the characteristics of the trial participants, the indication was revised to be restricted to subgroups (e.g., ESR1-mut population and level of PDL1 expression) as the newly proposed indication better reflected the studied population that may preferentially benefit from either enhanced efficacy or reduced toxicity compared with the extended population.
Later treatment line	Indication changes to the later treatment line are often based on the inclusion criteria of the pivotal study with respect to prior treatments or on subgroup analyses that demonstrated significant treatment effects. For oncology products, difficulties in isolating drug effects on hard efficacy end points, like PFS and OS, led to the establishment the utility of therapy for later treatment lines. Additionally, limited trial participants (small sample size) did not support reliable conclusions as to efficacy between treatment lines resulting in restricted indications. For ATMPs, such changes are usually related to safety profiles, if the therapy was known to have severe adverse events.
Change in characterizing the disease	A critical combination of clinical practice guidelines, similar biological and demographic characteristics of the targeted population, high unmet medical needs and expected similar treatment benefits gave rise to the need to revise the disease severity.
Expanding the population	The molecule was targeting the expression of a certain gene and all patients whose condition is due to that gene would benefit from the treatment.

### How the Indication Changes Could Impact the Eligible Population and JCA PICO

Five distinct scenarios were observed in terms of how indication changes would likely impact the eligible clinical population and corresponding JCA PICO (see Table 4): 1. Major impact: Central subgroup added, or replacing a central population; 2. High impact: Adding a subpopulation; 3. Moderate Impact: Excluding a central subgroup, 4. Minor impact: Excluding a subpopulation or a subgroup. Indication changes generally restricted the population to a smaller group, and the addition of a new population(s) was rare. In some cases, restricting the indication could also change the clinically eligible population to such a large extent that it would become essentially a different subgroup or subpopulation, which would constitute its own PICO completely. The most common example of this was when the indication was moved to a later treatment line.

**Table 4.** Indication changes observed, the impact on the eligible population and the risk to the JCA process

Risk to JCA	Scenarios	Examples observed
Major impact	Central subgroup added, replacing a central population	Later treatment line becomes the central population (*n* = 7). *“Relapsed or refractory” becomes “after 2 or more lines” (multiple products).* Population conditional on nonestablished prior therapies (*n* = 1). *“at least one line of endocrine therapy” becomes “at least one line of endocrine therapy including a CDK 4/6 inhibitor.” (Orserdu).*
High-impact	Subpopulation added	Broadening the disease definition to include subtypes (*n* = 1) *“…. vision loss due to Leber’s congenital amaurosis or retinitis pigmentosa inherited retinal dystrophy…” becomes “vision loss due to inherited retinal dystrophy” (Luxturna)*
Moderate impact	Central subgroup excluded	Limiting to more severe disease (*n* = 2). *“…locally advanced or metastatic” becomes “advanced” (Rybrevant).*
Minor impact	Subpopulation excluded	Clinical definition changes that excluded a small subpopulation (*n* = 1). *Added “symptomatic” and “femoral” (Spherox).*
	Subgroup excluded	Population conditional on a well-established prior therapy or genetic marker (*n* = 2). *Added “PD L1 expression < 1 percent” (Opdualaq).*

The most frequent indication changes in our assessment which was associated with a “major impact” risk for the PICO (18 percent, *n* = 8), was largely driven by the change to move to a later treatment line (16 percent, *n* = 7, Table 2). The other product (Orserdu) with a potentially “major impact” had two changes to its indication that specified a prior therapy and focused on a more severe patient group as shown in Table 2. One product (Luxturna) was associated with a “high impact risk” due to population broadening (Table 4). The remaining products with indication changes (12 percent, *n* = 5) were associated with a “minor to moderate impact” on the JCA process due to the fact that distinct populations or subgroups were excluded.

## Discussion

The JCA process is expected to create unpredictability and complexity for both health technology developers and HTA bodies. Particularly, HTA bodies are concerned about the potential changes between HTD-proposed and regulator-approved indications. This analysis demonstrates that in the majority of cases in our sample, the proposed eligible population has not changed during the regulatory process, despite rapporteur questions or major objections on the proposed indication being common. Only one-third had an actual difference between the original HTD-proposed and regulator-approved indication. The results on frequency of indication changes align with previous research in this area [7]. Given that the deliberation for the final indication is a process that only ends at the adoption of the positive opinion [13], information from regulatory rapporteurs during the regulatory process would not accurately predict likely changes to an indication, and consequentially reliably inform impacts on the EU HTA PICO. The greatest risk for the process would be to reduce the HTA PICO scope too early before the regulatory process has concluded, based on the list of questions.

### Managing and Mitigating the Risks of Needing a PICO Resurvey: A Considered Decision is Needed

Where the proposed and the final approved indication differed, the most common change was narrowing the proposed population to a specific subgroup. In situations whereby narrowing the population risks the introduction of new subgroups or subpopulations to the PICO, a delay mitigation strategy would be needed to avoid undermining the ultimate goal of the HTA regulation of faster patient access [2]. These results show that indication changes could be anticipated based on the pivotal evidence and patient characteristics in the trials, and so an active role for the HTD during the PICO process to share this knowledge would be a valuable risk management approach.

The other common impact is where content is no longer required. Only an administrative step would be needed in these instances; the PICO could be reissued, and the respective chapters removed from the dossier. There may be cases where this may not be straightforward, especially if data or assumptions are connected between the excluded groups and those remaining. In these instances, it would be beneficial for the HTD to inform the assessors of the impact this would have on dossier development.

It was rare for a broader population initially proposed by the HTD to be included in the indication, and where it was, it was unlikely to have been possible to anticipate. Therefore, a new PICO survey would likely be needed along with the implied subsequent delay. It may be beneficial in these scenarios to have a staggered option whereby the JCA could start for the initial population, whilst the PICO survey and dossier development for the new population are completed. This would also be warranted by the regulatory process because the applicant will have the option to choose whether to include the broader population or not, due to Market Authorization Holder (MAH) liability for the new population.

Where a product was moved to a later treatment line, there in principle could be changed to the comparators in the assessment scope. However, the way the current scoping process is proposed to be conducted, it would be likely that comparators for the later treatment lines would have been included due to differences in treatment landscapes between the Member States and the comparators they have proposed already through the initial PICO survey.

These results demonstrate that indication wording changes would not dictate that a PICO resurvey would definitively be needed, but that an active and considered decision should be made. The need for a PICO resurvey in this regard is low and could be further mitigated with comprehensive upfront scoping, for products at high risk.

### Managing Evidentiary Requirements: A Nuanced Decision for Delay

The JCA process has tight timelines for dossier development, and therefore small changes to the requirements, as determined by the PICO, can have a knock-on impact on the dossier development. However, a definitive need for a delay to enable dossier development or adaption was not identified in any of the scenarios observed, as there was always the potential for the HTD to have prepared for potential indication changes in advance. In all scenarios there was also a risk that the HTD could need time to adapt the dossier appropriately, depending on the nature of the changes applied. There may be cases where this may not be straightforward, especially if data or assumptions are connected between the excluded groups and those remaining. In all instances, it would be beneficial for the HTD to inform the assessors on the impact any PICO changes would have to dossier development; to avoid unnecessary delays, or setting the process up for failure because the dossier requirements could not be met within the timeframe.

### Recommendations

The Implementing Regulation on JCA outlines that individual assessors propose a way forward without input from the HTD, relying solely on the assessor’s assumption about the risks of indication changes and consequent PICO impact. Because any difference between the initial HTD-proposed and regulator-approved indication is only certain *after* the CHMP has adopted its opinion, reopening the assessment scope will take place relatively late in the process, after the JCA dossier is already submitted and the assessment has begun.

Reopening the scoping process is therefore potentially costly for all stakeholders; for developers and JCA assessors, there are resource costs, while for national level decision makers and patients, there are opportunity costs in terms of time to access. Distinct criteria are needed for making the decision to reopen the scope as indicated in Article 16 in the Implementing Regulation for JCA. The absence of a guideline on how to assess the potential PICO impact of an indication change may result in inconsistency across the system. A collaborative role for the HTD in the JCA scoping process would help mitigate the risks of delays in the JCA process associated with regulatory indication changes. This could be achieved through:

- HTD contribution to the initial PICO survey process, to ensure potentially critical subgroups from the trials are comprehensively included.
- HTD and JCA assessor dialogue throughout the scoping process, to identify the risk of an indication change, what it may be, and seek to mitigate the impact on the assessment.
- Communication between the HTD, European Commission and JCA assessors, at the time point when an indication change is confirmed. The purpose would be to assess whether there is a meaningful impact on the scope needed for the JCA, and dossier development, whether a PICO resurvey, or process delay(s) is needed.
  - Some distinct criteria for when a scope resurvey is needed would be valuable to support this discussion.
- A role for the HTD throughout the process is to support procedural decision-making processes, by being able to request and contribute rationale for a PICO resurvey, reissue, or a delay to the HTA process.
- HTDs should leverage the possibility for joint scientific consultations early on as well as the scoping explanation meeting to indicate if there is a risk that the assessment scope does not adequately cover the aspirational indication, based on the regulatory strategy, to avoid the need to reopen the assessment scope later in the process.

### Limitations

The study sample excluded products that were approved only conditionally. This decision was made due to the fact that conditionally approved products also go through a shorter deliberation process and may warrant an additional study to understand whether the faster deliberation process would result in different outcomes. The study assessed the PICO impact of an indication change through general patterns rather than a case-by-case analysis. The impact of indication changes on the eligible population relied on clinical assumptions and was not verified by clinical experts. The potential PICO impact was assumed and is dependent on the initial PICO survey and methodology for consolidating the final PICOs which was not publicly available at the time of research.

## Supporting information

Heikkinen et al. supplementary material Heikkinen et al. supplementary material

## Supplementary material

The supplementary material for this article can be found at http://doi.org/10.1017/S0266462324004641.

## References

[1] European Commission. Health Technology Assessment: Commission welcomes the adoption of new rules to improve access to innovative technologies; 2021, European Commission: European Union.

[2] *European Union Regulation on Health Technology Assessment*; 2021.

[3] *COMMISSION IMPLEMENTING REGULATION (EU)* *laying down, pursuant to Regulation (EU) 2021/2282 on health technology assessment, procedural rules for the interaction during, exchange of information on, and participation in, the preparation and update of Joint Clinical Assessments of medicinal products for human use at Union level, as well as templates for those joint clinical assessments* In: Commission E, editor. Brussels; 2024.

[4] European Medicines Agency, Wording of therapeutic indication. A guide for assessors of centralised applications C.f.H.M. Products, Editor. 2019, European Medicines Agency: European Union.

[5] Rohr, U.P., Iovine, M., Rudofsky, L., et al., A decade comparison of regulatory decision patterns for oncology products to all other non-oncology products among Swissmedic, European Medicines Agency, and US Food and Drug Administration. Clin Transl Sci. 2023. 16(9): p. 1569–1581. 37408165 10.1111/cts.13567 PMC10499418

[6] Willemsen, A., et al., EUnetHTA relative effectiveness assessments: Efforts to increase usability, transparency and inclusiveness. Int J Technol Assess Health Care. 2022; 38: p. e41. 35615861 10.1017/S0266462322000058

[7] Behring A. What impact will EU HTA have on the AMNOG process? In: Impact of EU HTA on the AMNOG procedure, E.B.f.I.P.o.B. Assessment, editor; 2023. Interdisciplinary platform on Benefit Assessment. p. 19–20.

[8] EUnetHTA21, PICO EXERCISE I - PLUVICTO 2023: Netherlands.

[9] EUnetHTA21, PICO EXERCISE II - EBVALLO TABELECLEUCEL 2023: Netherlands.

[10] EUnetHTA21, PICO EXERCISE III - POMBILITI 2023: Netherlands.

[11] EFPIA, EU HTA regulation for oncology medicines: Learnings from a simulation on the impact of proposed EUnetHTA21 methods. European Federation of Pharmaceutical Industries and Associations: Brussels; 2024.

[12] European Medicines Agency, Guidance document on the content of the <co>rapporteur’s day 80 critical assessment report for generic medicinal products (Article 10.1 only). Overview and list of questions – generic medicinal products C.f.H.M. Products, editor European Union; 2015.

[13] Bujar M et al., Transparency in European Medicines Agency and US Food and Drug Administration Decision Making: Is it possible to identify the Rationale for Divergences in Approved Indication From Public Assessment Reports? Clin Ther. 2021; 43:888–905. 33883070 10.1016/j.clinthera.2021.03.010

[14] Gemeinsamen Bundesausschusses, Stellungnahme der hauptamtlichen unparteiischen Mitglieder des Gemeinsamen Bundesausschusses (G-BA) vom 02.04.2024 zum Entwurf der “COMMISSION IMPLEMENTING REGULATION (EU) […] laying down, pursuant to Regulation (EU) 2021/2282 on health technology assessment, procedural rules for the interaction during, exchange of information on, and participation in, the preparation and update of joint clinical assessments of medicinal products for human use at Union level, as well as templates for those joint clinical assessments” und “ANNEXES I to III”. 2024.

[15] European Medicines Agency. Newsletters. European [cited 15 May 2024]; Available from: https://www.ema.europa.eu/en/news-and-events/publications/newsletters.

[16] European Medicines Agency, CAT quarterly highlights and approved ATMPs 2024.

