# Supplementary material for: Impact of indication changes on scoping for European Union Joint Clinical Assessment: scale of the problem and how to address it
Source: Int J Technol Assess Health Care. 2024 Nov 25;40(1):e62. doi: 10.1017/S0266462324004641 (PMC11703616; doi:10.1017/S0266462324004641)

**Supplementary Figure 1. The EU Joint Clinical Assessment process and the relevant linkage points with the EU centralized marketing authorization process**


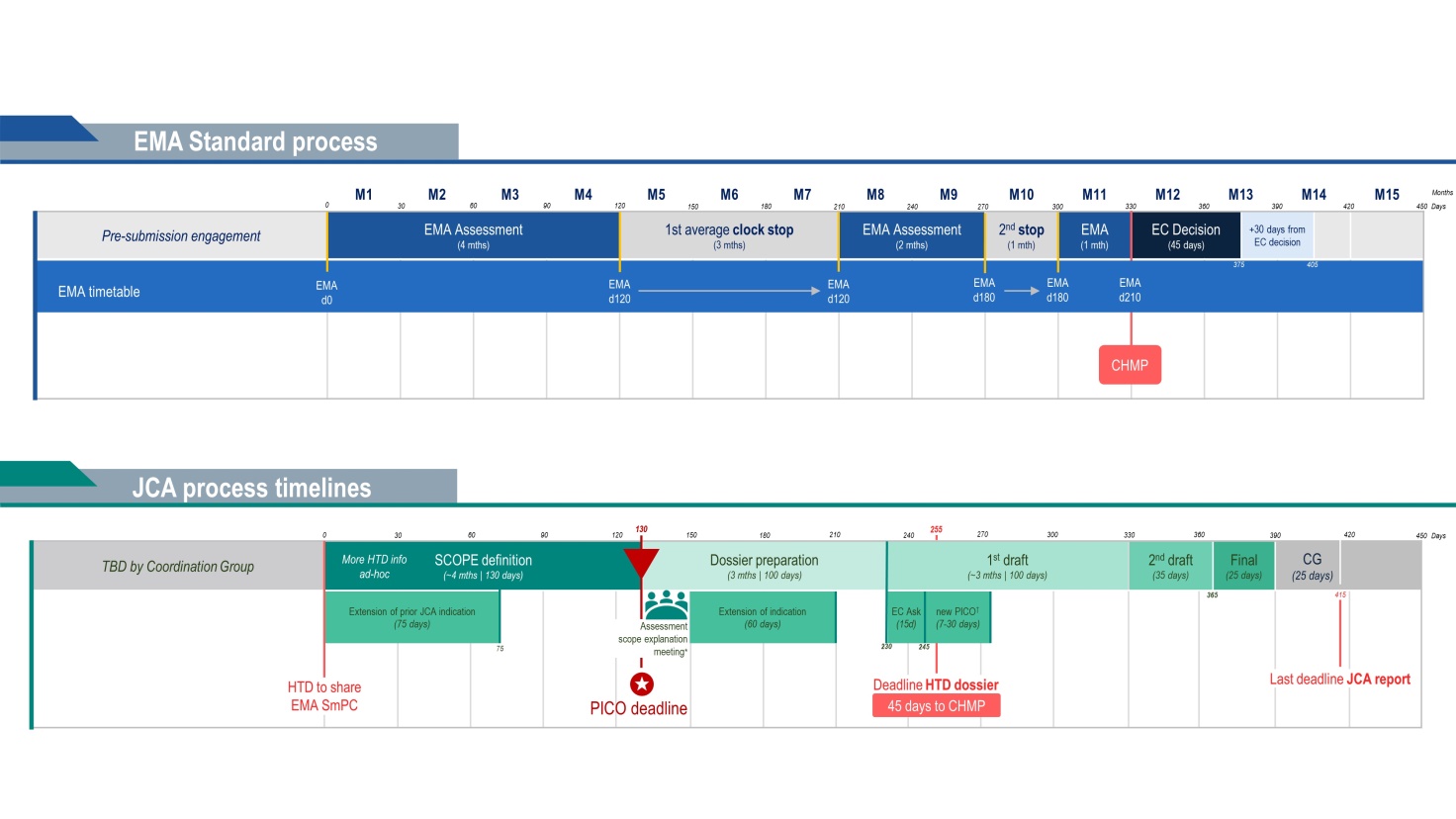

Supplement: Heikkinen et al. supplementary material [file S0266462324004641sup001.docx]
